# Continuing Professional Development – Radiation Therapy

**DOI:** 10.1002/jmrs.70059

**Published:** 2026-02-27

Maximise your continuing professional development (CPD) by reading the selected article and answering the five questions. Please remember to self‐claim your CPD and retain your supporting evidence. Answers will be available via the QR code and published in JMRS—Volume 73, Issue 4, December 2026.

## Stabilised Hyaluronic Acid Gel Rectal Spacers in MRI‐Guided Brachytherapy for Gynaecological Cancers: A Prospective Feasibility Study

Carminia Lapuz, Sylvia Hanna, Eddie Lau, Adeline Lim, Mark Tacey, Daryl Lim Joon, Claire Dempsey, Jenny Sim, Michael Chao, https://doi.org/10.1002/jmrs.70048.

1. Which of the following is a primary advantage of using stabilised hyaluronic acid for rectal spacer placement in gynaecological cancer brachytherapy?
  - Provides immediate structural stability by hardening right after injection
  - Fully absorbs within a few weeks, lowering the risk of fistula formation
  - Maintains spacing throughout the entire brachytherapy course due to extended resorption time
  - Minimises imaging interference by being less visible on ultrasound
2. According to the study, which anatomical entry point was most used for insertion of the rectal spacer into the rectovaginal space?
  - Perineum
  - Posterior vagina
  - Vaginal vault
  - Rectum
3. What was the mean target‐to‐rectum distance achieved with stabilised hyaluronic acid spacer insertion on the initial brachytherapy fraction?
  - 7.82 mm
  - 15.45 mm
  - 23.27 mm
  - 24.09 mm
4. Based on patient‐reported outcomes in the feasibility study, how many patients experienced pain or a sensation of heaviness related to rectal spacer placement?
  - 0
  - 1
  - 2
  - 3
5. How did clinicians evaluate the ease of incorporating rectal spacer insertion into the standard workflow for MRI‐guided gynaecological brachytherapy?
  - Very difficult
  - Slightly difficult
  - Neutral
  - Easy

### Recommended Further Reading

1. N. Murakami, K. Okuma, T. Kato, and H. Igaki. “Now Is It Time to Implement Spacers in Cervical Cancer Brachytherapy?” *Journal of Radiation Research* 63 (2022): 696–698, https://doi.org/10.1093/jrr/rrac031.

2. M. Svatos, E. Chell, D.A. Low, V. Pigrish, P.F. Orio, K. Miller, and M.T. King. “Symmetry, Separation, and Stability: Physical Properties for Effective Dosimetric Space with a Stabilised Hyaluronic Acid Spacer.” *Medical Physics* 51 (2024): 6231–6245, https://doi.org/10.1002/mp.17292.

3. C. Lapuz, M. Kain, M. Chao, D.L. Joon, C. Dempsey, and J. Sim. “Injectable Bio‐Absorbable Spacers in Brachytherapy for Gynecological Cancers: A Scoping Review.” *Journal of Contemporary Brachytherapy* 16 (2024): 467–477, https://doi.org/10.5114/jcb.2024.146834.

## Answers

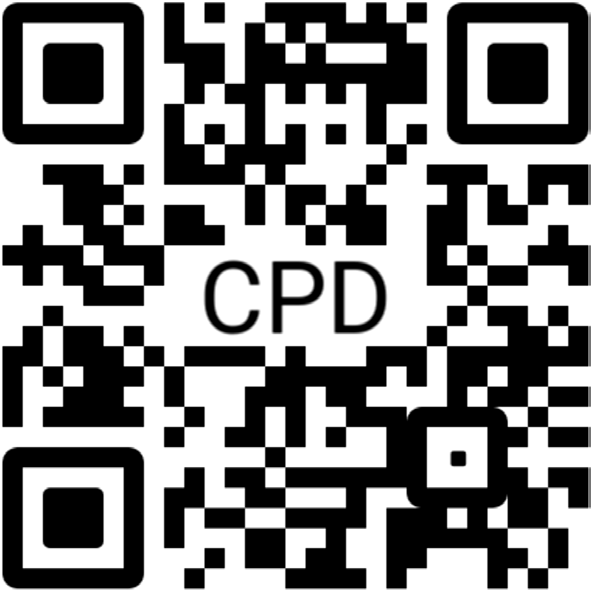

Scan this QR code to find the answers.

